# Theoretical Model for the Impact-Initiated Chemical Reaction of Al/PTFE Reactive Material

**DOI:** 10.3390/ma15155356

**Published:** 2022-08-03

**Authors:** Guancheng Lu, Peiyu Li, Zhenyang Liu, Jianwen Xie, Chao Ge, Haifu Wang

**Affiliations:** State Key Laboratory of Explosion Science and Technology, Beijing Institute of Technology, Beijing 100811, China; 3120195177@bit.edu.cn (G.L.); 1120181461@bit.edu.cn (P.L.); 3120215137@bit.edu.cn (Z.L.); 3120205130@bit.edu.cn (J.X.); gechao@bit.edu.cn (C.G.)

**Keywords:** reactive material, impact initiated chemical reaction, theoretical model

## Abstract

Reactive material (RM) is a special kind of energetic material that can react and release chemical energy under highly dynamic loads. However, its energy release behavior is limited by its own strength, showing unique unsustainable characteristics, which lack a theoretical description. In this paper, an impact-initiated chemical reaction model is proposed to describe the ignition and energy release behavior of Al/PTFE RM. The hotspot formation mechanism of pore collapse was first introduced to describe the decomposition process of PTFE. Material fragmentation and PTFE decomposition were used as ignition criteria. Then the reaction rate of the decomposition product with aluminum was calculated according to the gas-solid chemical reaction model. Finally, the reaction states of RM calculated by the model are compared and qualitatively consistent with the experimental results. The model provides insight into the thermal-mechanical-chemical responses and references for the numerical simulation of impact ignition and energy release behavior of RM.

## 1. Introduction

Reactive material, fabricated by pressing/sintering polymer matrix (typically polytetrafluoroethylene) and active metal powders, has metal-like strength and explosive-like reaction capability [1]. Due to its unique impact energy release characteristics, RM has been widely studied and has developed novel military applications over the past decades [2,3].

Significantly different from that of traditional energetic materials such as explosives and propellants, the energy release behavior of RM is closely related to its strength [4]. Under highly dynamic loads, RM is fragmented and scattered, and a local deflagrate reaction occurs. However, the chemical reaction cannot spread in the dense material [5], eventually causing the chemical reaction to be extinguished in the material. Therefore, the existing models for explosives and propellants are not suitable for characterizing the impact ignition and unsustainable chemical reaction process of RMs, and a new theoretical model is urgently needed.

Due to the complex impact ignition and the chemical reaction process of RM, experimental studies are widely used. For the impact ignition mechanism, Ames [6] suggested that material fragmentation is a prerequisite for chemical ignition, and the energy release behavior of RMs is determined by loading conditions, which is proved through vented chamber experiments. Mock [7,8] proposed a stress-delay time mechanism as an ignition criterion based on the Taylor impact test in a vacuum environment according to the first flare time under different loading conditions. On this basis, Ge C [9,10] further found that the impact ignition behavior of RM is related to the stress and strain rate of dynamic loading conditions. Recently, Tang Le [11] proposed that the shock ignition of RM starts from the local hotspot region based on the explosive loading test, and Jiang [12] calculated the relationship between impact ignition and porosity of RM based on the thermal behavior of plastic work generated by pore collapse. To describe the chemical reaction of RM, the Arrhenius equation based on the combustion rate measured by differential scanning calorimetry (DSC) is widely used to phenomenologically characterize the reaction process [13,14,15,16]. However, although this increasing understanding can be used for reference, the evolution that considers both the dynamic response and the energy release behavior of RM still lacks description using a theoretical model.

In this paper, the dynamic response and energy release process of RM is divided into two stages: impact ignition and chemical reaction. In the impact ignition stage, the local temperature rise of the material was calculated based on the hotspot theory of pore collapse, and the thermal decomposition rate of PTFE was calculated based on the hotspot temperature. Complete failure and fragmentation of the material were taken as the ignition criteria for reaction calculation. In the chemical reaction stage, the reaction rates of PTFE decomposition products and aluminum were calculated using the gas-solid chemical reaction model. According to the above analysis, a mechanical-thermal-chemical coupling model was developed to describe the impact-initiated chemical reaction of RM. Based on the data of inert numerical simulation, the reactive states of the RM rod were calculated using the model and compared with the experimental results. This research can provide a reference for further studies of numerical simulation on impact ignition and energy release characteristics of fluoropolymer-based RM.

## 2. Evolution from Impact Ignition to Chemical Reaction

The impact ignition and energy release behavior of RM is a complicated process of dynamic responses and chemical reactions. As experiments have revealed, the stable propagation of chemical reactions can only take place in a powder RM with a low density (less than 0.5 theoretical density) [5]. On the other hand, explosives [11] and lasers [17] cannot cause a sustainable chemical reaction in condensed RM, and only high-speed impact and fragmented materials lead to a wide range of reactions [18,19,20,21]. These results indicate that material fragmentation is a prerequisite for large-scale and sustainable chemical reactions. Therefore, in this paper, RM impact fragmentation was used as the criteria for reaction initiation, and the impact-initiated chemical reaction of RM was divided into two stages: impact ignition and chemical reaction, and the concept of the decomposition extent of PTFE was used in both stages.

In the impact ignition stage, the temperature rise induced by uniform plastic deformation of the material, could not heat the entire RM rod to a fire temperature or ignite the chemical reaction. Ames [6] proposed that some additional energy related to the crack propagation properties and the associated void collapse of RM is the key point to the ignition process. Cai [22] analyzed the pore compression and temperature rise of porous aluminum-rich PTFE/Al energetic materials under dynamic loads. They thought that during the compression process, the temperature rise of the RMs near the pores is mainly affected by the hole’s inner diameter and loading pressure. Since the RM is a void-rich compressive sintering composite, it is a reasonable ignition mechanism that the chemical reaction is initiated from local hotspots. 

In the chemical reaction stage, considering that the chemical reaction cannot directly take place between solid PTFE and Al, it is assumed that the chemical reaction occurs between the gaseous decomposition products of PTFE and the solid Al granules. It should be noted that the thermal decomposition process of PTFE has a variety of channels, among which the main channel is to generate CF_2_ gas in the absence of oxygen [23]. Therefore, it is assumed that all the thermal decomposition product of PTFE is CF_2_ gas, and the chemical reaction can be simplified to the combustion reaction between gas and solid reactants. 

**Figure 1:**
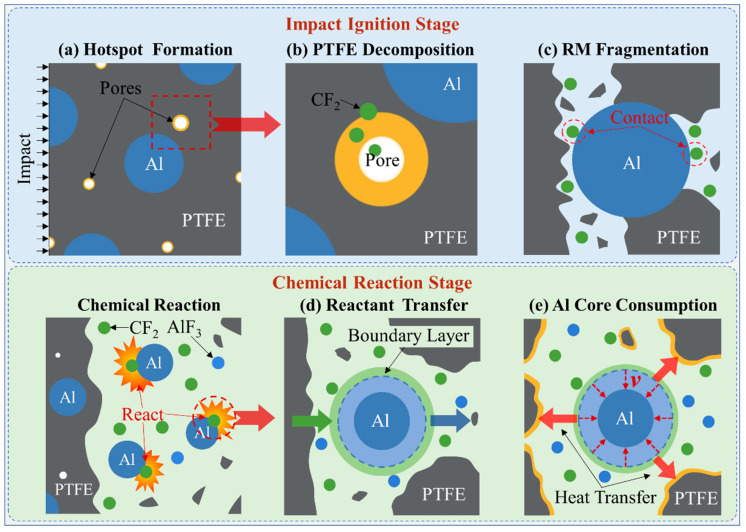
Typical impact ignition and chemical reaction process of RM.

Overall, a schematic of the typical impact-initiated chemical reaction process of RMs is shown in Figure 1. In the impact ignition stage, there are some pores inside the initial RM. Then, under highly dynamic loads, the material around the pores generates a local hot region (Figure 1a) due to the work of plastic deformation and collapse shear. The PTFE around the hotspot decomposes (Figure 1b) to produce oxidizing gas reactant CF_2_ after exceeding the threshold decomposition temperature. Subsequently, the material is fractured (Figure 1c) and the CF_2_ gas is released while exposing the aluminum particles to the gaseous reactant atmosphere and taking on a chemical reaction. In the chemical reaction stage, there is a reaction boundary layer on the surface of the solid aluminum particle. The decomposition product CF_2_ is transferred through the boundary layer to the surface of the aluminum reaction core as a reactant (Figure 1d). The Al reaction core is consumed at a consumption rate ν (Figure 1e), and the reaction product AlF_3_ flows out through the boundary layer. Then, the energy released by the chemical reaction will push up the temperature of the surrounding PTFE. If the temperature could exceed the decomposition temperature of PTFE (approximately 750 K from Ref. [15]), more CF_2_ gas would be released, thus providing enough gaseous reactants for further chemical reactions in the reaction region, and finally, all materials could react completely. Otherwise, the chemical reaction stops when all the CF2 gas is consumed. Next, we will establish theoretical models for the impact ignition stage and the chemical reaction stage of RM, respectively.

## 3. Impact Ignition Behavior of RM

### 3.1. Hotspot Formation Caused Temperature Rise

**Figure 2:**
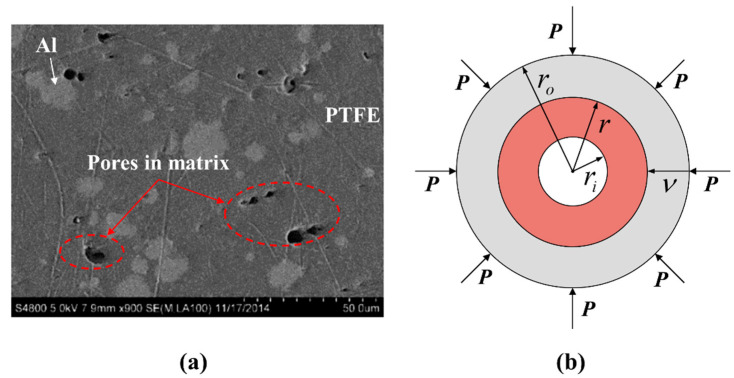
Image and schematic of pores inside the Al/PTFE microstructure: (**a**) SEM of Al/PTFE microstructures from Ref. [26] and (**b**) schematic of one-dimensional elastic-viscoplastic cavity collapse model.

To characterize the impact ignition behavior described above, the elastic-viscoplastic single spherical shell collapse model was adopted to describe the hotspots formation inside the RM during the dynamic loads. In the study of the explosive impact ignition problem, Kim [24,25] proposed that the temperature rise of materials around the hotspots is caused by mechanical deformation, heat conduction, and chemical reaction energy release. For fluoropolymer-based RMs, according to the SEM of PTFE/Al (73.5/26.5) RM [26] (shown in Figure 2a), the pores which will collapse under impact compression and form hotspots mainly appear inside the PTFE matrix. Meanwhile, in condensed RM without fragmentation, only extremely high temperatures (over 900 K [27]) can lead to direct reactions between PTFE and Al. Therefore, in the impact ignition stage, the calculation of the hotspots effect is assumed to exclude the energy released by chemical reactions and the mechanical deformation and heat transfer are counted for the temperature rise of the RM.

Figure 2b shows the schematic of the one-dimensional elastic-viscoplastic pore compression model where P is the periodic boundary condition, $r_{i}$ is the inner radius of the pore, $r_{o}$ is the outer radius of the spherical shell, and r is the current radius of a random position. At the beginning, the initial porosity of a cell is,

(1) $$\alpha =\frac{\rho_{t}-\rho }{\rho_{t}}=\frac{r_{i}{}^{3}}{r_{o}{}^{3}}$$

where $\rho_{t}$ is the theoretical density which is determined by the mass ratios of the components, and ρ is the actual density of the RM. According to Geng [28], the actual density of the RM prepared under the cold press-sintering process is related to the molding pressure and sintering temperature, and under ideal preparation conditions, the porosity of PTFE/AL (73.5/26.5) is about 5%. The collapse velocity of the spherical shell under uniform external pressure loading can be calculated as follows:

(2) $$\nu =\left[\frac{P-P_{g}}{4G\left(r_{o}^{-3}-r_{i}^{-3}\right)r^{2}}\right]\delta \left(t\right)+\frac{\gamma }{2\left(r_{o}^{-3}-r_{i}^{-3}\right)r^{2}\tau }\left[\left(P-P_{g}\right)H\left(t\right)-2\sqrt{3}\ln\frac{r_{o}}{r_{i}}\right]$$

where v is the motion velocity of the localized material, $P_{g}$ is the initial gas pressure in the pores, t is time, δ(t) is a delta function at t=0, H(t) is a step function, τ is the shear yield strength and $\tau =\sigma_{0}/\sqrt{3}$. G is the shear modulus, and G=0.0233 Mbar. γ is the adiabatic exponent, $\gamma =\gamma_{1}\sigma_{0}$, $\sigma_{0}$ corresponds to the static yield strength and $\gamma_{1}$ is constant. The motion velocity v can be used to calculate the compression state of pores. When the pores are completely closed, plastic compression can be continued as homogeneous materials, but hotspot-caused temperature rise is no longer calculated. On the other hand, when the material is fractured, the pore structure is destroyed and the calculation of hotspot-caused temperature rise stops. The temperature rise caused by mechanical deformation of spherical shell collapse is:

(3) $${\left(\frac{dT^{*}}{dt}\right)}_{M.D.}=\left(\frac{9}{4\rho_{PTFE}C_{p}}\right)\frac{{\left(P-P_{g}-2\sqrt{3}\tau \ln\frac{r_{0}}{r_{i}}\right)}^{2}}{{\left(r_{0}^{-3}-r_{i}^{-3}\right)}^{2}r^{6}}\cdot \frac{\gamma }{\tau }$$

where $T^{*}$ is the temperature of each position inside the spherical shell. The subscript M.D. represents the mechanical deformation, $\rho_{PTFE}$ and $C_{p}$ is the density and specific heat of PTFE, respectively. After mechanical deformation, there is a heat conduction process inside the material, so the total temperature change can be obtained as follows:

(4) $$\frac{dT^{*}}{dt}={\left(\frac{dT^{*}}{dt}\right)}_{M.D.}+\frac{1}{\rho_{PTFE}C_{p}}\frac{1}{r^{2}}\frac{\partial }{\partial r}\left(r^{2}k^{*}\frac{\partial T^{*}}{\partial r}\right)$$

where $k^{*}$ is the heat transfer efficiency of PTFE. The first term is the temperature rise caused by mechanical deformation and the second term is the temperature change caused by heat conduction. Then, the total temperature change in the spherical shell can be calculated by the following equation:

(5) $$T_{n+1}^{*}\left(r,t\right)=T_{n}^{*}\left(r,t\right)+\int_{t_{n}}^{t_{n+1}}\frac{dT^{*}}{dt}\left(r,t\right)dt$$

### 3.2. PTFE Decomposition Rate

Under highly dynamic loads, the PTFE around the pores is heated to the decomposition temperature and begins to depolymerize. According to the experimental measurement of He [29], the pyrolysis rate of spherical PTFE powders with average radii of 10 μm and 70 μm in the temperature range of 1400~3300 K is:

(6) $$W\left(T\right)=2.23\times 10^{3}\exp\left(-77.0/RT\right)s^{-1}$$

where R is the molar gas constant, and its value is 8.314 J/mol·K. Finally, the decomposition extent of PTFE in the hot spot stage can be calculated according to the temperature-time history:

(7) $$\Lambda_{n+1}=\Lambda_{n}+\frac{1}{M}\int_{r_{i}}^{r_{o}}\int_{t_{n}}^{t_{n+1}}2\pi r\rho \left(r\right)W\left(T\right)dtdr$$

where M is the total mass of the material in the spherical shell, r is the spherical coordinate radius, and ρ(r) is the density of each position in the shell.

**Figure 3:**
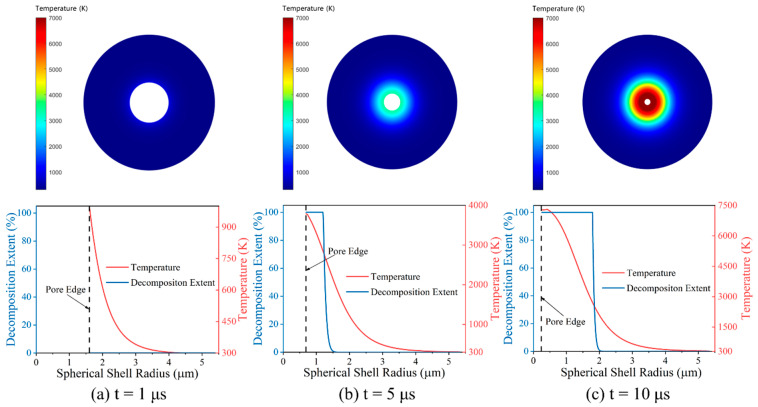
Typical temperature rise of pore collapse and decomposition extent of PTFE at 20 kbar constant pressure loads.

Figure 3 shows the typical temperature rise of pore collapse and the decomposition extent of PTFE at 20 kbar constant pressure loads. The results show that with the increase of time, the pores are compressed and their radius is continuously reduced. The high-temperature region inside the PTFE keeps expanding while the extent of fully decomposed PTFE keeps increasing. The decomposition extent of PTFE in the spherical shell was obtained by weighing the decomposition extent at all positions along the diameter direction. Assuming that all hotspots formed in RM under highly dynamic loads are similar, the characteristics of all hotspots in RM can be represented by studying the formation process of the single spherical shell collapse model described above. Therefore, the geometric parameters of the single spherical shell collapse model can be determined according to the physical parameters of the RM itself, the particle size, and density/porosity.

### 3.3. Ignition Criteria

According to the above analytical process, the decomposition extent of PTFE caused by hotspot temperature rise under highly dynamic loads is obtained. However, the decomposition products of PTFE around the hotspots cannot effectively contact the active aluminum particles, thus, material fragmentation is required to provide sufficient contact opportunities for reactants. In addition, some studies [30] have shown that the material crack tip also provides high temperature and further promotes the chemical reaction. Therefore, the ignition criteria of RMs can be summarized as follows:

1. PTFE depolymerizes to release gaseous reactant.
2. The material is fragmented.

The first criterion for the hotspot effect has been fully discussed in the two sections above. The second criterion concerns the dynamic response behavior of the RM, which has been studied extensively [10,31,32]. The Johnson-Cook strength model, which describes the strength behavior of materials subjected to large strains, high strain rates, and high temperatures, is employed for the RM in this paper. The model defines the yield stress as

(8) $$\sigma =\left(A+B\varepsilon_{p}^{N}\right)\left(1+C\ln{\dot{\varepsilon }}_{p}^{*}\right)\left(1-T_{H}^{m}\right)$$

where $\varepsilon_{p}$ is the effective plastic strain, ${\dot{\varepsilon }}_{p}^{*}$ is the normalized effective plastic strain rate, $T_{H}$ is the homologous temperature, $T_{H}=\left(T-T_{room}\right)/\left(T_{melt}-T_{room}\right)$, *A*, *B*, *C*, *N*, and *m* are five material constants. Raftenberg [31] determined the parameters for the 74 wt.% PTFE/26 wt.% Al RM experimentally.

After high-speed impact, the RM may suffer large deformation or even failure. The concept of failure means that the material can no longer withstand tensile loads and is often used to simulate the ejection behavior of fractured debris. The Johnson-Cook failure model is often used to model ductile failure of materials experiencing large pressures, strain rates, and temperatures. It consists of three independent terms that define the dynamic fracture strain as a function of pressure, strain rate, and temperature.

(9) $$\varepsilon^{f}=\left(D_{1}+D_{2}e^{D_{3}\sigma^{*}}\right)\left(1+D_{4}\ln{\dot{\varepsilon }}^{*}\right)\left(1+D_{5}T_{H}\right),D=\sum \frac{\Delta \varepsilon }{\varepsilon^{f}}$$

where Δε is the increment of effective plastic strain, $\varepsilon^{f}$ is the failure strain, σ* is the mean stress normalized by the effective stress, and *D*_1_, *D*_2_, *D*_3_, *D*_4_, and *D*_5_ are constants. The failure accumulation factor *D* is incremented and stored as the ratio of the effective fracture strain. When *D* < 1, the material is assumed to be intact. Once *D* = 1, the failure occurs and the material is assumed to be fractured, then the calculation can turn into the chemical reaction stage.

## 4. Impact-Initiated Chemical Reaction of RMs

### 4.1. Transfer Efficiency of Gaseous Reactants

The hotspots formed when the pores inside the material were subjected to impact compression and shearing, which led to a sharp increase of internal energy in the PTFE matrix around the pores, and soon the matrix temperature heats up to the decomposition temperature of PTFE. The PTFE matrix depolymerizes and produces CF_2_, CF_3_, and other gas products [23]. When the material is fragmented after impact loading, the gaseous decomposition products contact with Al particles and undergo a chemical reaction. Under oxygen-free conditions, the overall reaction process between PTFE and Al is as follows [33]:

$$C_{2}F_{4}\Rightarrow 2CF_{2}\left(g\right)$$

$$2CF_{2}\Rightarrow CF\left(g\right)+CF_{3}\left(g\right)$$

$$3C_{2}F_{4}+4Al\left(s\right)\Rightarrow 4AlF_{3}\left(g\right)+6C\left(s\right)$$

We assume the solid aluminum particles are surrounded by gaseous decomposition products of PTFE when calculating the combustion reaction process. Therefore, the overall combustion reaction of RM can be regarded as a gas-solid two-phase flow reacting around several spherical Al particles.

**Figure 4:**
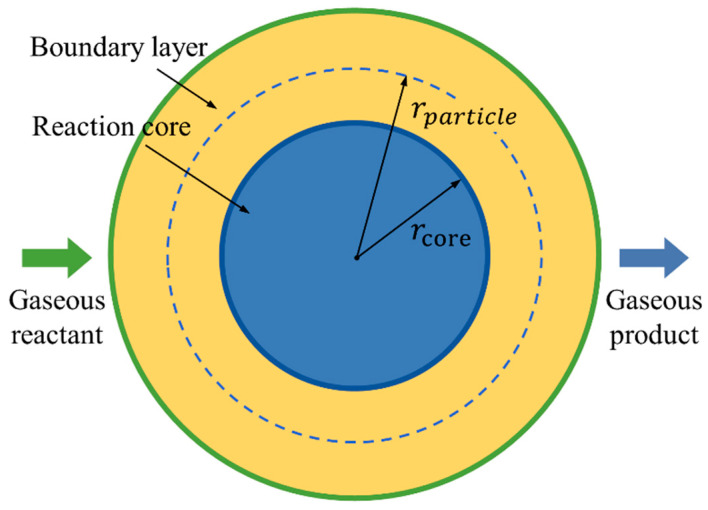
Gas-solid chemical reaction process in RM.

As shown in Figure 4, there is a chemical reaction boundary layer between the two reactants, and only when the gaseous decomposition products of PTFE cross the boundary layer and reach the surface of Al particles can they undergo a chemical reaction. Hence the mass transfer process of gaseous reactant through the boundary layer needs to be calculated first. Considering that the gas environment and the gas flow around aluminum particles are relatively limited, the boundary layer mass transfer can be regarded as the diffusion process of gas molecules from high concentration to low concentration. Then the dimensionless relation of gas boundary layer mass transfer theory is used to describe the mass transfer process between a single particle and gas [34]:

(10) $$Sh=2.0+0.6Re^{\frac{1}{2}}Sc^{\frac{1}{3}},Re=0\sim 200$$

$$Sh\equiv \frac{2h_{D}r_{particle}}{D_{T}}$$

(11) $$Re\equiv \frac{2\rho_{g}u_{g}}{\mu }$$

$$Sc\equiv \frac{\mu }{\rho_{g}D_{T}}$$

where Sh is the Sherwood constant, Re is the Reynold constant, Sc is the Schmidt constant, $h_{D}$ is the mass transfer efficiency, $\rho_{g}$ is the gas density, and $u_{g}$ is the airflow velocity. Both Sh and Sc need to be calculated using diffusion coefficient $D_{T}$, which can be estimated using Chapman-Enskog empirical formula:

(12) $$D_{T}=0.001858T^{\frac{3}{2}}\frac{{\left(\frac{1}{M_{CF_{2}}}+\frac{1}{M_{AlF_{3}}}\right)}^{\frac{1}{2}}}{P_{out}\sigma_{AB}^{2}\Omega_{AB}}$$

where T is the gas Kelvin temperature surrounding the Al particle, $M_{CF_{2}}$ and $M_{AlF_{3}}$ are the relative molar masses of gaseous reactants and gaseous products, respectively. $P_{out}$ is the pressure of the principal part of the gas phase (Bar), $\sigma_{AB}$ is the average collision radius, and $\Omega_{AB}$ is the collision integral (0.417 from ref. [34]). The mass transfer efficiency can be expressed by combining the Equations (10)–(12),

(13) $$h_{D}=\frac{D_{T}}{r_{particle}}\cdot \frac{1+a{\left(\frac{r_{core}}{r_{particle}}\right)}^{0.5}}{\frac{r_{core}}{r_{particle}}}$$

where $r_{core}$ is the current Al core radius, $r_{particle}$ is the original Al core radius, and $a=0.3S_{c}{}^{1/3}R_{e}{}^{1/2}$. In this model, the decomposed gas environment is relatively closed, and the airflow velocity can be approximated to $u_{g}=0$, then $R_{e}=0$ and a=0. Thus, the mass transfer efficiency $h_{D}$ can be converted into:

(14) $$h_{D}=0.001858T^{\frac{3}{2}}\frac{{\left(\frac{1}{M_{CF_{2}}}+\frac{1}{M_{AlF_{3}}}\right)}^{\frac{1}{2}}}{P_{out}\sigma_{AB}^{2}\Omega_{AB}r_{core}}$$

### 4.2. Aluminum Core Consumption Rate

When the PTFE decomposition products were transferred to the surface of aluminum particles, the gas-solid chemical reaction began to take place. According to the quasi-steady state hypothesis, the reaction rate in this chemical reaction depends only on the substance concentration on the particle surface. The consumption rate of the incoming material is much faster than the gaseous reactants transfer efficiency, which means that the gaseous reactants transferred into the reaction zone will be consumed in time, so as to achieve a steady-state:

(15) $$kC_{CF_{2}core}=h_{D}(C_{CF_{2}out}-C_{CF_{2}core})$$

where k is the reaction rate constant, and according to the Arrhenius equation, $k=Ae^{-\frac{Ea}{RT}}$. $C_{CF_{2}}$ represents the concentration of gaseous reactants, and the subscripts out and core represent the principal part of the gas phase outside the boundary layer and the surface of the Al reaction core, respectively. The CF_2_ concentration outside the boundary layer can be calculated as follows:

(16) $$C_{CF_{2}out}=\frac{2\Lambda_{PTFE}m\omega_{PTFE}M_{CF_{2}}}{VF+m\omega_{PTFE}\left(F-\Lambda_{PTFE}\right)/\rho_{PTFE}}$$

Here m is the unit mass, $\omega_{PTFE}$ is the mass fraction of PTFE, V is the unit volume and *F* is the reaction content. During the reaction process, the size of the Al core continuously shrinks, so the reaction rate can be characterized by the linear velocity of the Al core interface (the consumption velocity of the Al core along the diameter direction):

(17) $$-\frac{\rho_{particle}}{4}\cdot \frac{dr_{core}}{dt}=kC_{CF_{2}core}$$

Here, $\rho_{particle}$ is the aluminum particle density. Combining the Equations (15) and (16),

(18) $$-\frac{dr_{core}}{dt}=\frac{4C_{CF_{2}out}/\rho_{particle}}{1/h_{D}+1/k}$$

The total combustion reaction rate is defined as follows,

(19) $$R=\frac{dF}{dt}=\frac{dF}{dr_{core}}\frac{dr_{core}}{dt}$$

where *R* is the reaction rate and there is a relationship between *F* and $r_{core}$:

(20) $$F=1-{\left(\frac{r_{core}}{r_{particle}}\right)}^{3}$$

$$\frac{dF}{dr_{core}}=-\frac{3}{r_{particle}}{\left(\frac{r_{core}}{r_{particle}}\right)}^{2}=-\frac{3}{r_{particle}}{\left(1-F\right)}^{\frac{2}{3}}$$

Substituting Equations (18) and (20) into Equation (19), it can be obtained:

(21) $$R=\frac{12C_{CF_{2}out}}{r_{particle}\rho_{particle}}\cdot {\left(1-F\right)}^{\frac{2}{3}}\cdot {\left(\frac{r_{particle}P_{out}\sigma_{AB}^{2}\Omega_{AB}}{0.001858T^{1.5}{\left(\frac{1}{M_{CF_{2}}}+\frac{1}{M_{AlF_{3}}}\right)}^{\frac{1}{2}}}\cdot \frac{\sqrt[{3}]{1-F}}{1+a{\left(1-F\right)}^{\frac{1}{6}}}+\frac{1}{A}e^{\frac{Ea}{RT}}\right)}^{-1}$$

where $E_{a}$ is the activation energy and its value is $50.836\;\mathrm{kJ}\cdot {\mathrm{mol}}^{-1}$ [11]. Finally, Equation (21) is the reaction rate of the combustion reaction between PTFE decomposition products and Al under highly dynamic loads.

## 5. Validation of Impact-Initiated Chemical Reaction Model for RMs

### 5.1. Calculation Process

**Figure 5:**
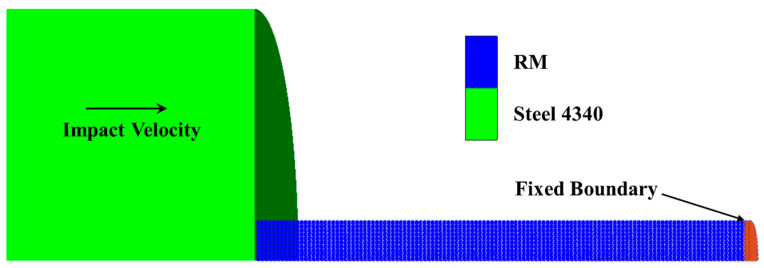
Scheme of simulation model.

The impact-initiated chemical reaction model for RMs was calculated based on the simulation results of the inert collision behavior of the RM rod. Mock [7,8] performed experiments to investigate the impact ignition of the RM rods impacted by steel anvils in a vacuum. To simulate the inert dynamic response of RM to mechanical shock, the Smooth Particle Hydrodynamics (SPH) method was adopted to develop the finite element models. As shown in Figure 5, the finite element model consists of a RM rod (φ 7.6 mm × 50.8 mm) and a steel anvil (φ 50 mm × 25.4 mm), and a quarter symmetric model was used to shorten the computation duration. The RM rod was constructed using 0.38 mm diameter SPH particles, and the 1 mm × 1 mm Lagrange cell was adopted for the steel anvil.

In this paper, the shock equation of state (EOS) is used to describe the behavior of RM and steel. In the Autodyn program, the shock EOS is established from the Mie–Gruneisen form of EOS based on shock Hugoniot,

(22) $$P=P_{H}+\Gamma \rho \left(E-E_{H}\right)$$

where it is assumed that $\Gamma \rho =\Gamma_{0}\rho_{0}=\mathrm{constant}$ and

(23) $$P_{H}=\frac{\rho_{0}c_{0}u\left(1+u\right)}{{\left[1-\left(s-1\right)u\right]}^{2}}$$

(24) $$E_{H}=\frac{1}{2}\frac{P_{H}}{\rho_{0}}\left(\frac{u}{1+u}\right)$$

here, $\Gamma_{0}$ is the Gruneisen coefficient, $u=\left(\rho /\rho_{0}\right)-1$, ρ is the current density, $\rho_{0}$ is the initial density, s is a linear Hugoniot slope coefficient, and $c_{0}$ is the bulk sound speed. The Johnson–Cook strength and failure model, which is the form in Equations (8) and (9), is used to represent the strength and failure behavior of RM. The main material model parameters with the basic units of cm, g, and μs for RM used in the simulation are listed in Table 1 and Table 2. The parameters of RM are from reference [10,11,31] and the parameters of steel 4340 are from the Autodyn material libraries.

After calculation based on the simulation model, the pressure-time history data of RM for each selected time were obtained from the Autodyn program using the print function [35]. These data were input into Equations (3)–(5) as loading conditions to calculate the hotspot formation induced temperature rise. Then the PTFE decomposition extent was calculated through Equation (7). It should be noted that at the same time as calculating the decomposition extent, Equation (2) is used to calculate the pore compression velocity. When the pore is closed, the calculation of the hotspot stage stops even if the material does not reach a failure state. Temperature, pressure, volume, and mass of RM particles at the failure time (failure factor *D* reaches 1) are obtained through numerical simulation as input variables in the calculation of the chemical reaction process.

For the chemical reaction calculation, the decomposition extent $\Lambda_{PTFE}$ can be used to obtain the concentration of the decomposition product $C_{CF_{2}out}$ according to Equation (16). By substituting these variables into Equation (21), the reaction content of RM with time can be calculated iteratively. It should be noted that the temperature used in calculating the chemical reaction rate R is the average temperature of the material particle. This is because the chemical reaction occurs on the surface of Al particles, while the heat transfer efficiency of Al particles is much higher than that of PTFE, leading to the result that the temperature can be evenly distributed to the whole Al particle instantaneously. Therefore, the average temperature of the whole particle is used as the reference temperature in the chemical reaction stage, and it is assumed that the energy released by the chemical reaction is converted to temperature only based on the initial specific heat of the RM.

### 5.2. Calculation Results for Impact Ignition Behavior

**Figure 6:**
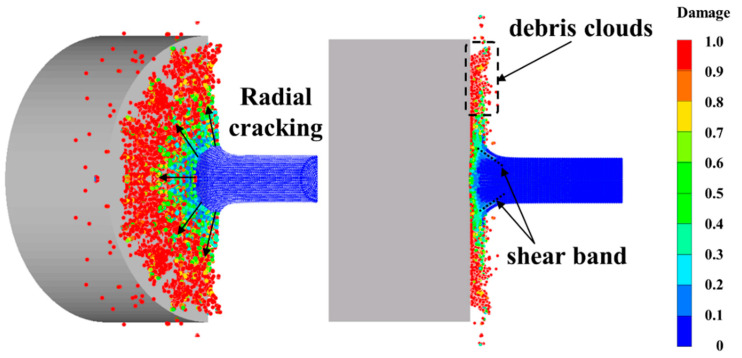
Simulation result of RM rod with impact velocity of 775 m/s at 30 μs.

A typical simulation result of a RM rod at 30 μs and impact velocity of 775 m/s is shown in Figure 6. After being impacted by the target plate, the top area of the RM rod is deformed and thickened to a “mushroom” shape. Radial cracking occurs at the edge of the mushroom part of the RM rod. The outer materials completely fail (D = 1), and are extruded from the mushroom and dispersed outward, forming debris clouds. At the same time, the radially expanding mushroom involves circumferential shear bands inside the RM rod, which will result in the subsequent failure of nearby materials.

**Figure 7:**
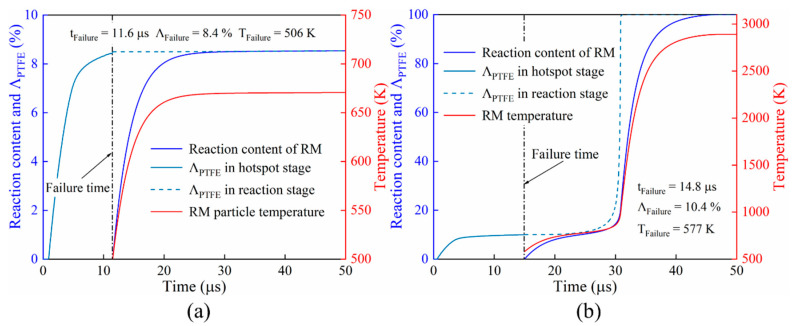
Reaction content profiles of two typical kinds of reacting particles: (**a**) partially reacted particle, and (**b**) completely reacted particle. Note: $t_{Failure},\;\Lambda_{Failure},\;T_{Failure}$ represent failure time, PTFE decomposition extent, and particle average temperature at the failure time, respectively.

Based on the results of the numerical simulation, the pressure-time history, failure time, and other parameters of each particle can be obtained. These parameters were substituted into the impact-initiated chemical reaction model, and the chemical reaction process of each particle was calculated. The profiles of typical completely reacted and partially reacted particles are shown in Figure 7. It can be seen from the results that the two particles accumulated a certain PTFE decomposition extent due to the hotspot effect before particle failure. After the complete failure of the material, the chemical reaction of the two particles occurred and pushed up the average temperature of the particles.

However, the partially reacted particle (Figure 7a) accumulated a lower PTFE decomposition extent as well as the average temperature (temperature rise induced by plastic work from the compression of the uniform particles from the simulation) at the failure time. After the failure of the material, the average temperature of the particle (a) can only increase to approximately 660 K due to the energy released from the chemical reaction. Because the temperature cannot maintain the further decomposition of PTFE, the chemical reaction stops. The decomposition extent of PTFE and the average temperature accumulated by a particle (Figure 7b) at the hotspot stage were higher because the particle failed later and the energy released by the initial chemical reaction pushed up the particle average temperature to above the PTFE decomposition temperature. Thus, the chemical reaction was sustained, and finally, all the material reacted completely.

**Figure 8:**
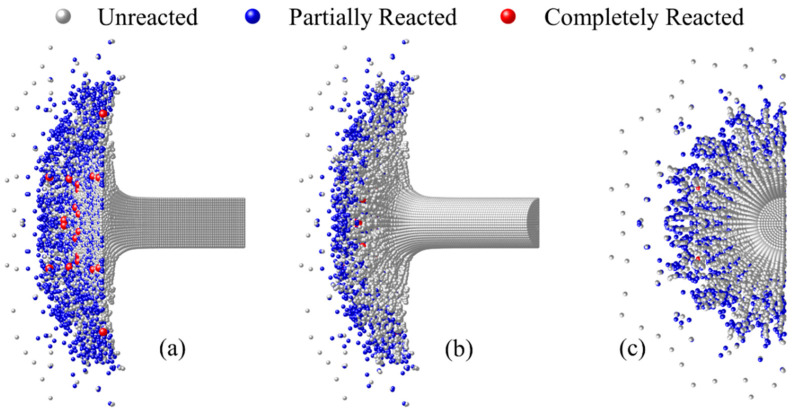
Images of RM rod from different perspectives at 775 m/s impact velocity and 30 μs: (**a**) cross-section view with a rotation angle of 30°; (**b**) surface view with a rotation angle of −30° and (**c**) top view.

The chemical reaction content of all particles in the RM rod was calculated, and the images of the RM rod from different perspectives at 30 μs and impact velocity of 775 m/s are shown in Figure 8. In the figure, the particles are painted gray, blue, and red, which represent the materials that were unreacted, partially reacted, and completely reacted, respectively. To show the reaction states more clearly, different rotation angles are used to present the images.

As can be seen from Figure 8a, the hotspot reaction mainly occurred on the contact surface between the RM rod and the steel anvil. With the radial diffusion of the RM, a large amount of partially and completely reacted particles appeared at the contact surface. However, from Figure 8b,c, radial cracking occurs at the surface of the RM rod, resulting in petal-shaped cracks on the surface material. Meanwhile, compared with the materials on the contact surface of the steel anvil, partially/completely reacted particles on the surface of the RM rod are greatly reduced. This is because the surface material of the RM rod suffered from low loading intensity and only a few particles failed, so almost no reaction occurs.

**Figure 9:**
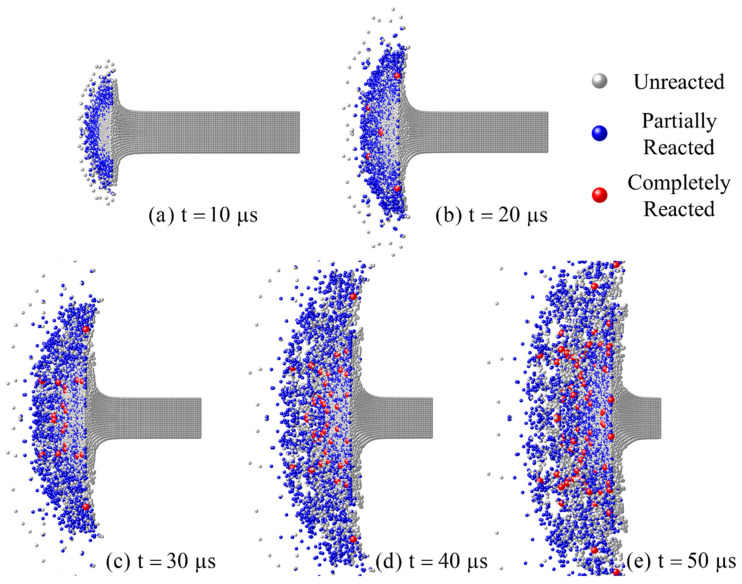
Reaction morphologies (cross-section view with a rotation angle of 30°) of RM rods with the impact velocity of 775 m/s at different times.

The reaction morphologies of the RM rods at different times are shown in Figure 9. As can be seen from the figures, with the continuous compression of the steel anvil, the top area of the RM rod gradually thickened to a mushroom shape. Then the radial cracking spread in the mushroom-shaped rod, and internal material fragmented and extruded, forming the radial debris clouds. The chemical reaction mainly takes place where the material has broken up and is flying outward. At 20 μs, completely reacted particles occur, and as time goes on, the number of partially reacted materials keeps increasing as well as the completely reacted ones. After 40 μs, although the material rod is further fragmented, the number of reactive particles changes little and becomes more dispersed. This is because some materials cannot completely fail and react, so the number of particles that can ignite a chemical reaction is limited.

In general, the chemical reaction began at the contact surface between the RM rod and the steel anvil. The material from the outer ring of the mushroom-shaped part of the RM rod extruded and a hotspot reaction first appeared. Then, fully reacted particles began to appear in the radial expansion part. As time goes on, chemical reactions took place at various locations on the contact surface between the RM rod and the anvil.

**Figure 10:**
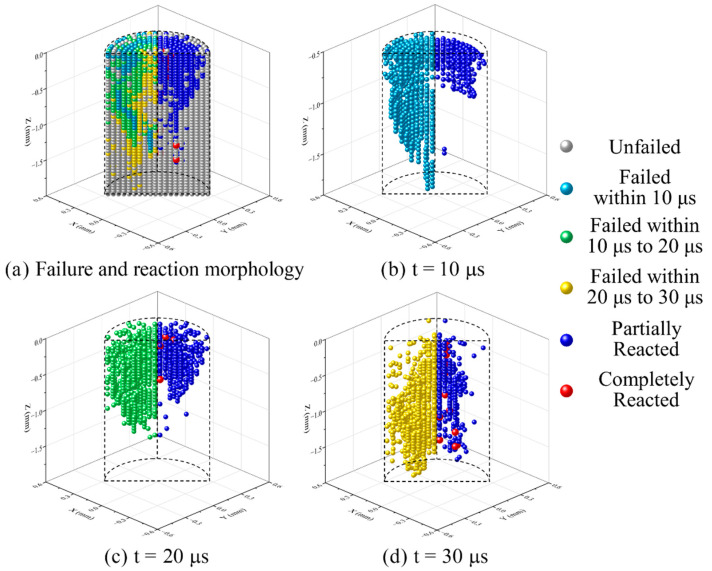
Failure and reaction morphology of the front third part of RM rod of 775 m/s impact velocity at different times: (**a**) overall failure image at 30 μs and corresponding reaction morphology at 60 μs; (**b**) particles failed within 10 μs; (**c**) particles failed within 10 μs to 20 μs; (**d**) particles failed within 20 μs to 30 μs.

To further analyze the failure and chemical reaction process of the RM rod under highly dynamic loads, the particles with different failure times and corresponding reaction states at an impact velocity of 775 m/s were plotted. As shown in Figure 10, the particles with different failure times are all compared with the corresponding reaction states of 60 μs. This is because the chemical reaction falls behind the failure of the material in time, and the duration of the complete chemical reaction is approximately 30 μs according to the result of Figure 7b. At the same time, only the front third part of the RM rod was cut for morphology to highlight the failure and reaction characteristics since the materials of other parts of the RM rod have not failed at 30 μs.

As shown in Figure 10a, the material that failed was first located in the shear band of the RM rod. During the impact loading process, these particles (wathet blue) first reach their tensile rupture strain. Then the green particles on both sides of the shear band were further compressed, reaching a failure state. At the same time, the RM rod was compressed into a mushroom shape, and the material on the outer surface also showed radial cracking, but failed particles only appeared at the cracks of the outer surface. Subsequently, the yellow particles continued to be compressed until they were extruded by the subsequent material and reached the failure state.

In terms of the chemical reaction, the material in the shear band failed the earliest, but the loading duration was short, so only partially reacted particles occurred, but no completely reacted particles were observed. For the subsequently failed material, only part of the hotspot reaction occurred near the outer surface of the rod, and completely failed particles appeared near the core of the rod. Finally, the failed core material accumulated the most PTFE decomposition extent at the hotspot stage, and produced the most completely reacted particles after failure because of the long duration and highly dynamic loads. This indicates that the particles which can partially and completely react need to meet sufficient loading intensity and duration to achieve a higher reaction content.

### 5.3. Comparison with Experiment

**Figure 11:**
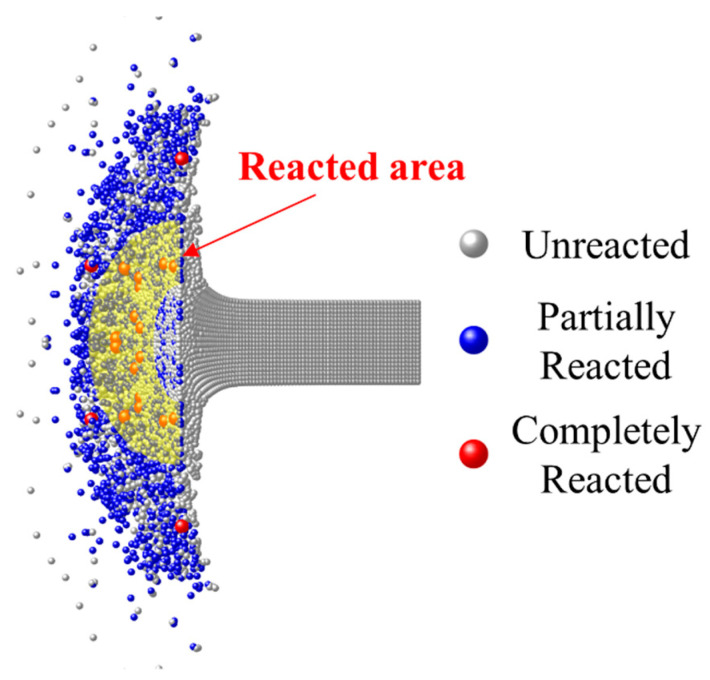
Calculated result (cross-section view with a rotation angle of 30°) at 775 m/s impact velocity and 30 μs (corresponding to the experimental result of the same loading conditions in Figure 4a of Ref. [8]).

To validate the impact-initiated chemical reaction model, the calculation results are compared with the vacuum collision test of Mock [8]. As shown in Figure 11, at the impact velocity of 775 m/s and 30 μs, the RM rod fractured under the impact loads, creating a scattering cloud of debris. In the experimental result of the same loading conditions in Figure 4a of Ref. [8], the part near the RM rod in the debris cloud showed obvious reaction light, and the impact light gradually weakened from the rod to the periphery. In the calculation results, the hotspot reactions occurred in a large number of the extruded material in the mushroom-shaped region of the RM rod. Many completely reacted particles appeared in the annular region close to the rod (yellow area in Figure 11), while only a few particles completely reacted in the outer ring.

This is because the material in the top area of the RM rod was the first to be crushed under dynamic loads. Although the load strength was high, the loading duration was short, and material failure occurred before the hotspot reaction temperature accumulation, so the complete reaction could not be achieved. For the materials subsequently loaded, the loading strength and duration are enough to ignite hotspot reactions and achieve a more adequate chemical reaction. Overall, for the macroscopic phenomenon, the chemical reaction fire gradually diminishes from the rod to the periphery, which is very consistent with the experimental results.

**Figure 12:**
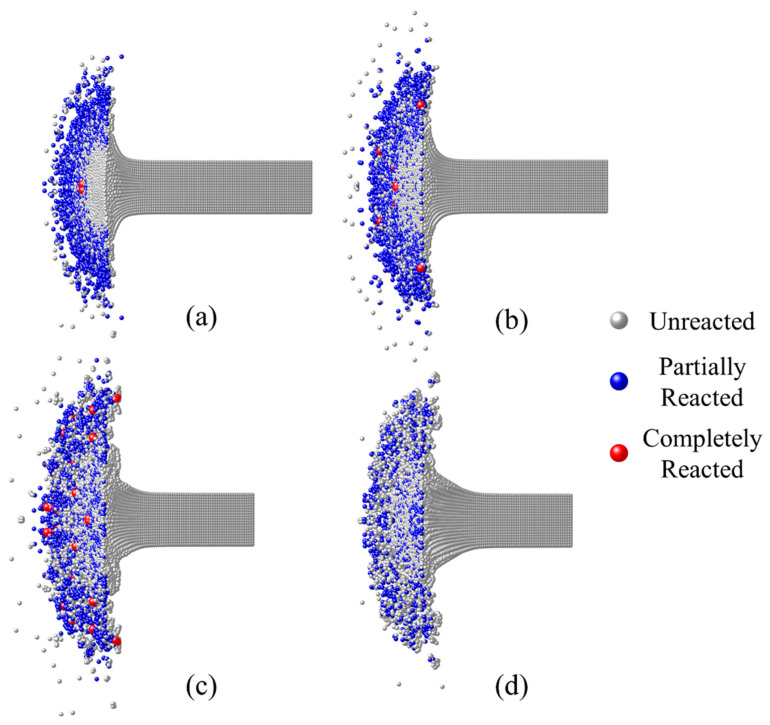
Reaction morphology of RM rod at the time for the first light of different impact velocities: (**a**) 969 m/s at 14 μs; (**b**) 775 m/s at 22 μs; (**c**) 617 m/s at 38 μs; (**d**) 468 m/s at 50 μs (no light).

Figure 12 shows the reaction morphologies of the RM rod at the time of the first light of different impact velocities in the experiments. In the case of higher velocity, the first light appeared earlier, and the mushroom-shaped part of the RM rod was smaller in size. The partially reacted particles were mainly concentrated in the outer region of the debris cloud, and the completely reacted particles appeared at the contact surface of the steel anvil and near the core of the rod. With the decrease in impact velocity (Figure 12a–c), the length of the compression part of the RM rod increased when the first light was observed, and the distribution of partially reacted materials in the debris cloud became more diffuse. This is because lower impact velocity corresponds to lower loading strength, while fewer materials will be ignited at the same time. When the fire light is observed, the material has accumulated a certain reaction content. Therefore, the lower the impact velocity is, the longer the loading time (until the time for the first light) will be, and the more completely reacted particles appear in the reaction morphology. However, when the impact velocity is further reduced (Figure 12d), the loading intensity will be insufficient, leading to no completely reacted particles in the material.

**Figure 13:**
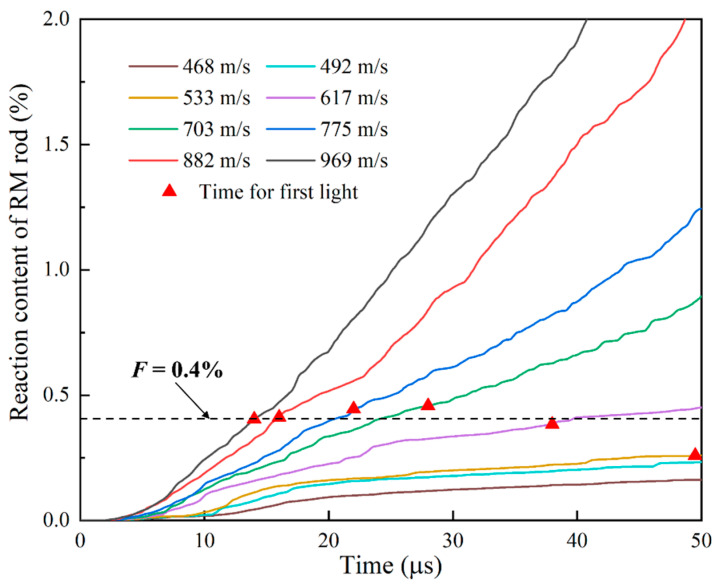
Reaction content of RM rods at different impact velocities.

The relationship between the reaction content and time of all particles in the RM rod at different impact velocities was statistically weighted according to mass, and the results are shown in Figure 13. The results suggest that with the increase of impact velocity, the time of the beginning of reaction content accumulation in materials is slightly earlier, and the reaction content increases successively. This is because, under different dynamic loads, RM particles will completely fail and ignite hotspot reaction, so the reaction content of the material accumulates to a certain degree. However, these reactions are too weak to be observed through macroscopic phenomena. When the total reaction content of the RM rod continues to accumulate to a certain extent, fully reacted particles begin to appear, leading to a higher probability of macroscopically visible firelight.

Assume that the overall reaction content of the RM rod with macroscopically visible flame is 0.4%, as shown in Figure 13. The red marks represent the time for the first light from the experiments. The result indicates that the higher the impact velocity is, the earlier the material accumulates to the threshold chemical reaction content, and the higher the probability of observing firelight in the macroscopic phenomena will be. This is qualitatively consistent with the experimental results. However, it should be pointed out that the calculation in this paper is based on inert collision simulation and does not consider that debris clouds will be more dispersed after the chemical reaction occurred. At the same time, the chemical reaction transfer between adjacent material particles is not considered, so the overall reaction content calculated is low.

## 6. Conclusions

In this paper, an impact-initiated chemical reaction model for Al/PTFE reactive material is proposed. Different from the phenomenological numerical model, the model can well characterize the impact of the unsustainable reaction behavior of RMs and can provide a reference for a numerical simulation of the impact ignition and energy release behavior of fluoropolymer-based RM. The main conclusions are as follows:

(a) Based on the evolution from impact ignition to chemical reaction, the PTFE decomposition and material fragmentation were chosen as the impact ignition criteria. The hotspot formation mechanism of pore collapse was introduced to describe the temperature rise as well as the decomposition process of PTFE. The reaction rate equation was established based on the gas-solid chemical reaction model.
(b) The decomposition products accumulated before the material fragmentation contact with Al particles and ignite the chemical reaction. The energy released by the initial chemical reaction pushes up the material temperature. When the material temperature exceeds the PTFE decomposition temperature, PTFE continues to decompose and react until the material is completely consumed. Otherwise, the chemical reaction stops, causing the RM to show unsustainable chemical reaction characteristics.
(c) The material which can completely react needs to meet sufficient loading intensity and duration. The material in the shear band of the RM rod failed earliest, but the loading duration was short, hence only partially reacted particles occurred. The failed core material accumulated the most PTFE decomposition extent at the hotspot stage and produced the most completely reacted particles after the material fragmentation because of the long loading duration.
(d) Based on the numerical simulation of the inert dynamic response of RM, the chemical reaction process of the Taylor rod is calculated using the model in this paper. The results are compared and qualitatively consistent with the experimental ones.

## Figures and Tables

**Table 1:** Material model parameters for RMs.

Hotspot Stage		Chemical Reaction Stage	
$\sigma_{0}$(Mbar)	1.95 × 10^−6^	$R_{g}$(J/mol·K)	8.314
$\gamma_{1}$	13	$\sigma_{AB}$(Å)	4.35
$\rho_{PTFE}$(g/cm^3^)	2.23	$\Omega_{AB}$	0.417
$k^{*}$(cm/μs·g)	2.40 × 10^−14^	$C_{p}$(cm^2^/μs^2^·K)	1.20 × 10^−5^

**Table 2:** Strength and failure model parameters for RMs.

A (Mbar)	B (Mbar)	N	C	m
8.044 × 10^−5^	2.506 × 10^−3^	1.8	0.4	1
D1	D2	D3	D4	D5
0.02	0.807	−1.873	−0.0392	−0.488

## Data Availability

Not applicable.
